# Variation in Chronic Automated Red Cell Exchange Practices for Sickle Cell Disease: Insights Into Isovolemic Hemodilution Use

**DOI:** 10.1002/jca.70146

**Published:** 2026-06-18

**Authors:** Jennifer M. Jones, Fatima Aldarweesh, Nicole Aqui, Aleh Bobr, Patricia Brunker, Mischa L. Covington, Juliana Guarente, Robert Hagar, Matthew Karafin, Divjot Singh Lamba, Grace Lee, Wen Lu, Ethan A. Mack, Gagan Muthur, Saadiya Nazli, Halima Olaniyan, Chinelo P. Onyenekwu, Jay S. Raval, Divya Setya, Yvette C. Tanhehco, Mamie Thant, Angelica Vivero, Yanyun Wu, Edward C. C. Wong, Nalan Yurtsever, Yan Zheng, Patricia A. Shi, Jennifer Webb

**Affiliations:** ^1^ Department of Pathology and Internal Medicine University of Michigan Ann Arbor Michigan USA; ^2^ University of Chicago Chicago Illinois USA; ^3^ Hospital of the University of Pennsylvania Philadelphia Pennsylvania USA; ^4^ University of Nebraska Medical Center Omaha Nebraska USA; ^5^ Massachusetts General Hospital Boston Massachusetts USA; ^6^ Brigham and Women's Hospital Boston Massachusetts USA; ^7^ Thomas Jefferson University Hospital Philadelphia Pennsylvania USA; ^8^ University of California San Francisco Benioff Children's Hospital San Francisco California USA; ^9^ University of North Carolina Chapel Hill North Carolina USA; ^10^ Post Graduate Institute of Medical Education and Research (PGIMER) Chandigarh India; ^11^ Duke University Medical Center Durham North Carolina USA; ^12^ Mayo Clinic Rochester Minnesota USA; ^13^ Columbia University Irving Medical Center New York New York USA; ^14^ University of California Irvine Irvine California USA; ^15^ Indiana University School of Medicine Indianapolis Indiana USA; ^16^ Department of Pathology and Laboratory Medicine University of Wisconsin‐Madison Madison Wisconsin USA; ^17^ Robert Larner, M.D. College of Medicine, University of Vermont Burlington Vermont USA; ^18^ Yashoda Hospital Ghaziabad India; ^19^ Department of Medicine, Division of Hematology‐Oncology University of Cincinnati Cincinnati Ohio USA; ^20^ Hoxworth Blood Center, University of Cincinnati College of Medicine Cincinnati Ohio USA; ^21^ Miami Miller School of Medicine Miami Florida USA; ^22^ Quest Diagnostics Chantilly Virginia USA; ^23^ Yale University School of Medicine New Haven Connecticut USA; ^24^ St Jude Children's Research Hospital Memphis Tennessee USA; ^25^ Seattle Children's Hospital Seattle Washington USA; ^26^ Department of Pediatrics and Pathology, George Washington University School of Medicine and Health Sciences Children's National Hospital Washington District of Columbia USA

**Keywords:** automated red blood cell exchange, practice variability, sickle cell disease, survey

## Abstract

Prior surveys of chronic automated red blood cell exchange (RCE) for patients with sickle cell disease (SCD) have identified considerable procedural variability, especially with the use of isovolemic hemodilution red blood cell exchange (IHD‐RCE). We conducted a survey of chronic RCE practices among American Society for Apheresis (ASFA) members to identify opportunities for practice harmonization and future studies. The ASFA SCD Research Subcommittee developed a 72‐item survey of chronic RCE practices, with a focus on IHD‐RCE. The survey was validated internally and distributed by email to all ASFA members from September 2024 to February 2025. One survey response from each institution was retained for data analysis. Descriptive statistics were performed. Sixty‐two survey responses, including three from international institutions, were retained for analysis. Sixty‐one percent of respondents (38/62) reported using at least one fixed RCE target, with end hematocrit being the most common fixed parameter (40%, 25/62), followed by fraction of cells remaining (31%, 19/62) and hemoglobin S% (27%, 17/62). Most respondents (60%, 37/61) performed IHD‐RCE at their institution, though other details such as the minimum hematocrit, exclusion criteria, and response to adverse effects were variable. Consistent pre‐ and post‐RCE laboratory monitoring practices were reported. Consistent with earlier surveys, procedural variability in chronic RCE practices for patients with SCD is observed. This highlights opportunities to compare outcomes where practice variability exists. Consensus guidelines based on institutional practices with optimal outcomes should be developed.

## 1 Introduction

Chronic transfusion therapy (CTT) remains a foundational treatment for patients with sickle cell disease (SCD) who experience persistent symptoms or progressive end‐organ dysfunction despite pharmaceutical therapies. Rigorous clinical studies have demonstrated that CTT reduces the incidence of stroke in high‐risk children with SCD and reduces stroke recurrence in children with SCD who have suffered an initial stroke [1, 2]. These results have been extrapolated to support the use of CTT for other complications of SCD in individuals of all ages, though evidence for these practices is generally low‐grade or based on consensus [3, 4]. When CTT is used to prevent SCD complications, the patient is often maintained at a goal hemoglobin S percentage (HbS%) of 30%–50%. CTT occurs via three modalities: simple transfusion, partial manual exchange transfusion, or automated red cell exchange (RCE) transfusion [5]. For individuals receiving CTT, the American Society of Hematology (ASH) recommends using RCE over other modalities to minimize iron loading, but no further guidelines are specified regarding the technical aspects of the apheresis procedure [6].

Isovolemic hemodilution red cell exchange (IHD‐RCE), a modification of standard RCE intended to reduce red cell utilization, lacks sufficient evidence to guide its use. When IHD‐RCE is performed, a hemodilution step occurs before RCE, wherein the apheresis device removes patient red blood cells (RBCs) to achieve a pre‐specified minimum hematocrit and continuously replaces the removed RBC volume with either saline or 5% albumin. Once the nadir hematocrit is reached, the exchange portion of the procedure occurs with donor RBCs to achieve a target post‐procedure fraction of cells remaining (FCR) and hematocrit. Kim et al. first described the IHD‐RCE procedure in the early 1990s, but recognition of the benefits of IHD‐RCE to reduce RBC usage did not occur until 2011 with the report by Sarode et al. [7, 8] Subsequent studies have confirmed RBC savings with IHD‐RCE; however, limited data inform the selection of safe minimum and post‐procedure hematocrit goals or appropriate candidates to undergo IHD‐RCE [9].

RCE practices for patients with SCD and the technical specifics of the procedure differ considerably between institutions. In 2018, Karafin et al. surveyed 99 American Society for Apheresis (ASFA) member institutions for their acute and chronic RCE practices and found marked variability in indications beyond treatment and prophylaxis of stroke and acute chest syndrome, goal HbS% values, and use of IHD‐RCE, among other factors [10]. In recent years, ASH and ASFA have published additional guidelines to address transfusion therapy in patients with SCD, though many questions remain regarding optimal technical settings for the RCE procedure [3, 6]. To update and expand the survey results from Karafin et al., we conducted a survey of ASFA member institutions to capture CTT practices using chronic RCE with a focus on IHD‐RCE. We sought to identify areas of practice variability that may benefit from further study or consensus that could drive harmonization of policies and procedures.

## 2 Materials and Methods

This study received exempt status from the Children's National Hospital Institutional Review Board as participation was anonymous and voluntary. Members of the ASFA SCD research subcommittee developed a 72‐item survey to record chronic RCE practices including indications for IHD‐RCE (Table S1). Survey responses were collected and managed using Research Electronic Data Capture (REDCap), an electronic data capture tool hosted at Children's National Hospital [11, 12]. Branching logic was incorporated into the survey design to enable respondents to bypass questions that were not applicable to their institution. Prior to distribution, six subcommittee members beta‐tested the survey to ensure appropriate functionality and to identify questions requiring clarity.

The survey was available to all ASFA provider members from September 2024 to February 2025, with three completion reminders sent to encourage participation.

### 2.1 Data Analysis

To avoid over‐representation from a single center, one survey response from each institution was retained for data analysis. If multiple responses from a single institution were received, the most complete response was included. If each response was equally complete, the first response received was included.

Descriptive statistics were performed. All categorical variables are presented as number of affirmative survey responses divided by the total number of survey respondents who answered the specific question, along with the corresponding percentage. Denominators were occasionally variable due to unanswered survey questions. Continuous variables are presented as medians and interquartile ranges (IQR). Univariate analysis was performed to determine if institutional characteristics, such as total number of patients in the program, correlated with procedural choices. *p*‐values less than 0.05 were considered significant. All data analysis was performed in Microsoft Excel (Microsoft Corp., Redmond, WA) and Stata/BE Version 18.0 (StataCorp LLC, College Station, TX).

## 3 Results

The survey was distributed to 415 ASFA members. The initial response rate was 17% (69/415) before excluding seven incomplete surveys or surveys from individuals working at the same institution (7/69, 10%). Sixty‐two unique respondents completed the survey. Thirty‐eight respondents identified their institution. Of these, 35 represented U.S. institutions, and three represented international institutions in three countries: Norway, India, and Lebanon. The median number of patients receiving RCE at each institution was 15 (IQR: 8–30). Most institutions served both adult and pediatric patients (34/62, 56%) followed by adults‐only (22/62, 36%).

Most respondents (39/61, 64%) identified as the Director of Apheresis services at their institution. Two respondents identified as the Director of the Sickle Cell Center. Ninety‐two percent of respondents (55/60), including one Director of the Sickle Cell Center, were responsible for setting RCE parameters. Years of experience were evenly distributed between 0 and 3 to greater than 20 years (Table S2).

### 3.1 Patterns for Setting Chronic RCE Parameters

Sixty‐one percent (38/62) of respondents reported setting fixed RCE parameters for all patients, with some respondents selecting multiple fixed RCE parameters. End hematocrit was the most common fixed parameter (25/62, 40%). The median fixed end hematocrit goal was 30% (IQR 30% to 30%). Respondents who used a fixed end hematocrit were likely to have a fixed HbS% target (OR 8.93, 95% CI: 2.43 to 32.82, *p* = 0.001). Five respondents who do not use fixed parameters described strategies to individualize end hematocrit goals (Table 1). Two providers set the end hematocrit to mirror the patient's pre‐transfusion hematocrit. Of all providers surveyed, including those who did and did not use fixed parameters, 87% (54/62) reported that the pre‐procedure hematocrit influenced the target end hematocrit in some way.

**TABLE 1:** Strategies to individualize chronic RCE parameters.^a^

“…If the indication is for stroke, tend to be more aggressive (FCR < 30%, HbS < 30%). If the indication is VOC only, we may set the to FCR 30–50%. The target Hct is set “closer” to the starting Hct to prevent iron overload”
“We vary the target from patient to patient. The pre‐procedure HbS is between 30%–50%; 30% is most common. Post‐procedure target Hct varies from 28% to 35%, but 30% is most common.”
“Target Hct is based on the pre‐procedure Hct: if the presenting Hct is < 25%, the goal is 27%. If 26%–29%, the goal is 30%. If 30%–32% the goal is the presenting Hct. If > 33%, the goal will be the presenting Hct minus 3%.”
“We set individual post‐procedure HbS and Hct goals, and target pre‐procedure HbS goals”
“Hct set to equal pre‐exchange Hct”

Abbreviations: FCR, fraction of cells remaining; HbS, hemoglobin S%; Hct, hematocrit; VOC, vaso‐occlusive crisis.

^a^ Grammatical errors are corrected for clarity.

Thirty‐one percent of respondents (19/62) used a fixed‐target FCR, with a median FCR of 30% (IQR 30 to 30%). One program described their strategy to individualize FCR targets based on indication for RCE (Table 1). Twenty‐seven percent of respondents (17/62) used fixed HbS% targets, with the median HbS% being 25% (IQR 20 to 30%). Five respondents (5/62, 8%) used both fixed FCR and HbS% targets. There was no correlation between using a fixed FCR and HbS% target (OR 0.92, 95% CI: 0.21–3.12, *p* = 0.9).

Use of fixed RCE parameters was not correlated with the total number of patients receiving chronic RCE in a program, pediatric or adult designation, respondent years of experience, or respondent title (Table S3).

### 3.2 Periprocedural Laboratory Studies

**FIGURE 1:**
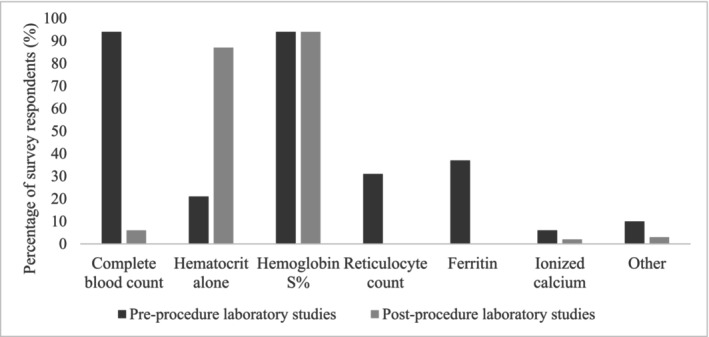
Laboratory studies performed prior to and following automated red blood cell exchange (*n* = 62 total survey respondents). Complete blood count (94%), hemoglobin S% (94%), serum ferritin (37%), and reticulocyte count (31%) were the most performed pre‐procedure laboratory studies. Hemoglobin S% (94%) and hematocrit (87%) were the most performed post‐procedure laboratory studies. “Other” pre‐procedure studies included comprehensive metabolic panel, basic metabolic panel, urinalysis, lactate dehydrogenase, and the direct antiglobulin test, and post‐procedure studies included “acid/base” status and liver function tests.

Survey respondents reported laboratory studies drawn routinely pre‐ and post‐RCE (Figure 1). The most common pre‐procedure studies were complete blood count and HbS% (58/62, 94% for both), serum ferritin (23/62, 37%), and reticulocyte count (19/62, 31%). Most providers (43/62, 69%) used the pre‐procedure HbS% to set the RCE interval. Approximately half of providers (32/62, 52%) used this value to set the FCR for the current procedure. Of those who did not use the pre‐procedure HbS% to set the FCR for the current procedure, 23% (14/62) used this value to set the FCR for future procedures. Most respondents obtain a post‐procedure HbS% (58/62, 94%) and hematocrit (54/62, 87%).

The pre‐procedure HbS% and hematocrit were obtained three or fewer days prior to the RCE procedure for 74% (46/62) and 84% (52/62) of respondents, respectively. A smaller percentage of respondents (6/62, 10%) collected samples for these studies on the day of the procedure. Most programs (37/62, 60%) did not report a HbS% turnaround time, but of those who did, 68% (17/25) received HbS% results within 3 days of sample collection.

### 3.3 Circuit Priming

Respondents who performed RBC priming (46/62, 74%) cited low pre‐procedure hematocrit (37/46, 80%), body weight (33/46, 72%), and high extracorporeal volume relative to total blood volume (TBV) (32/46, 70%) as RBC prime indications. More than half of respondents (38/62, 61%) did not use albumin as a custom fluid for priming when performing RCE. For those who performed albumin priming, the most common reasons were TBV percentage of the extracorporeal volume (18/24, 75%), body weight (17/24, 71%), and low pre‐procedure hematocrit (16/24, 67%).

### 3.4 Isovolemic Hemodilution (IHD‐RCE)

Most respondents (37/61, 60%) performed IHD‐RCE at their institutions (Table 2). Reducing donor exposures and iron overload were the most common reasons for using IHD‐RCE. Determination of the minimum hematocrit for the hemodilution phase of the procedure was variable, though most practitioners used a fixed, absolute decrease from baseline hematocrit (20/37, 54%). Only respondents from combined programs (i.e., pediatric and adult programs) targeted a minimum hematocrit of 21% (median, IQR 21%–24%; range 21%–24%). The maximum absolute decrease from baseline hematocrit was 5% (median, IQR 3%–5%; range 3%–5%) for pediatric‐only programs and 6% for adult‐only and combined programs (median, IQR 5%–6%; range 4%–6%, and median 5%–8%; range 5%–8%, respectively).

**TABLE 2:** Isovolemic hemodilution use characteristics (*n* = 36).^a^

	Adults (*n* = 11)	Pediatrics (*n* = 3)	Combined (*n* = 22)
*Reasons for performing, no. (%)*			
To save money using fewer units	3 (27)	—	9 (41)
Reduce donor exposures	11 (100)	—	18 (82)
Lower FCR for RBC units available and increase exchange interval	3 (27)	1 (33)	8 (36)
Decrease iron overload	6 (55)	3 (100)	14 (64)
Decrease procedure time	—	—	1 (5)
Other^b^	1 (9)	—	1 (5)
*Minimum hematocrit determination, no. (%)*			
Absolute lower limit	1 (9)	—	8 (36)
Relative % drop from baseline	4 (36)	—	3 (14)
Absolute decrease from baseline	4 (36)	3 (100)	13 (59)
Other^c^	3 (27)	—	5 (23)
Minimum hematocrit, %, median (IQR)	—	—	21 (21–24)
Maximum relative hematocrit drop, %, median (IQR)	6 (3–8)	—	15 (4–25)
Maximum absolute hematocrit drop, %, median (IQR)	6 (5–6)	5 (3–5)	6 (5–8)
*Do not perform IHD‐RCE in the following, no. (%)* ^d^			
Moyamoya syndrome	6 (18)	3 (12)	—
Silent cerebral infarctions	8 (24)	5 (20)	—
Mean pulmonary artery pressure > 25 mmHg	5 (15)	2 (8)	—
Left ventricular ejection fraction < 40%	14 (42)	8 (32)	—
GFR < 60 mL/min/1.73 m^2^	5 (15)	2 (8)	—
Other^e^	8 (24)	3 (12)	—
*All are eligible for IHD‐RCE, no. (%)* ^d^	9 (27)	5 (20)	—
Earliest days after stroke to start IHD‐RCE, days, median (IQR)	30 (1–180)	29 (0–180)	—

Abbreviations: FCR, fraction of cells remaining; GFR, glomerular filtration rate; Hct, hematocrit; IHD‐RCE, isovolemic hemodilution; RBC, red blood cell.

^a^ One program indicated use of IHD‐RCE but did not identify the age of patients treated. This program was excluded from analysis.

^b^ To reduce the number of RBCs used or per hematology request on a case‐by‐case basis.

^c^ Variable depending on pre‐procedure hematocrit (2, 5%), combination of relative percentage drop and lower limit threshold (5, 14%), hematologist request (1, 3%).

^d^ Branching logic within the survey permitted different responses for adult and pediatric patients, enabling combined programs to give different answers for each group. Therefore, combined program responses are incorporated into adult and pediatric categories respectively for these sections.

^e^ Baseline hematocrit < 23%–27% (7, 21%), history of myocardial infarction/cardiac dysfunction (3, 9%), history of ischemic stroke (3, 9%), pregnant status (2, 6%), acute indication (2, 6%), history of seizure (1, 3%), first year receiving chronic automated red blood cell exchange (2, 6%), and per hematology request (2, 6%).

Respondents avoided performing IHD‐RCE in adult and pediatric patients with a left ventricular ejection fraction of less than 40% (14/33, 42%; and 8/25, 31%), silent cerebral infarctions (8/33, 24%; and 5/25, 20%), and moyamoya syndrome (6/33, 18%; and 3/25, 12%) most commonly. Other cited reasons were baseline hematocrit less than 23%–27%, history of ischemic stroke within 3 years, and pregnancy. In contrast, 27% of adult providers (9/33) and 20% of pediatric providers (5/25) consider all patients eligible for IHD‐RCE, which was a selectable survey choice.

**FIGURE 2:**
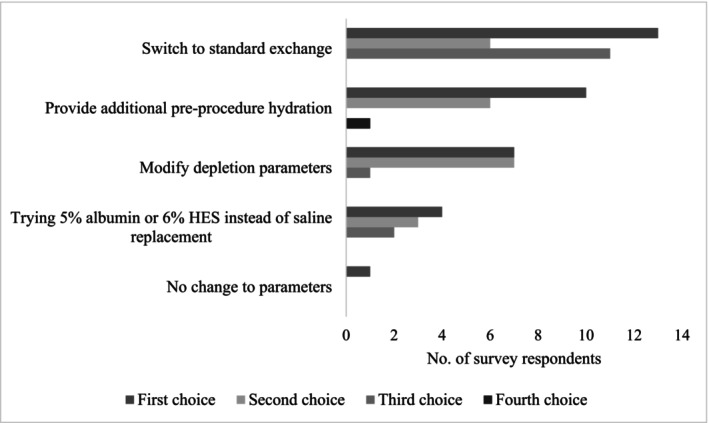
Corrective actions for IVH adverse effects. Thirty‐five survey respondents reported procedural adjustments for patients who develop IHD‐RCE‐associated symptoms. The most common first‐choice strategies were to switch to standard exchange and provide additional pre‐procedure hydration. The second most common mitigation strategy was to modify depletion parameters. Attempting IHD‐RCE using 5% albumin or 6% hydroxyethyl starch replacement during the hemodilution phase was chosen less frequently, and one respondent reported making no changes to the IHD‐RCE procedure if symptoms are reported. IHD‐RCE, isovolemic hemodilution; HES, hydroxyethyl starch.

Like standard RCE, IHD‐RCE is associated with adverse effects such as vasovagal symptoms, nausea, and malaise [8]. For patients who developed these symptoms, switching to standard RCE and providing additional pre‐procedure hydration were the most common first‐tier strategies to manage symptoms, included by those who ranked one or multiple strategies (Figure 2). Of those who ranked multiple strategies (22/35, 63%), providing additional pre‐procedure hydration and modifying depletion parameters ranked above switching to standard RCE, which was the most common third choice.

### 3.5 Other Procedural Considerations

No guidelines inform the use of SCD‐specific disease‐modifying therapies in combination with RCE. Our survey evaluated concurrent use of hydroxyurea, crizanlizumab, and voxelotor (Table S4). Of note, this survey was distributed prior to the voluntary market withdrawal of voxelotor in September 2024 [13]. Hydroxyurea was prescribed to a median of 50% (IQR 20%–80%) of patients on RCE to increase hemoglobin F% (44%), decrease vaso‐occlusive events (19%), and as a continuation from the pre‐transfusion therapy period (16%). Voxelotor and crizanlizumab were prescribed less often (median 0% [IQR 0%–1%] for both). For all disease‐modifying therapies, survey respondents noted that the decision to use these medications in addition to RCE was at the discretion of the treating hematology team, not exclusively those providing RCE.

### 3.6 RBC Unit Considerations

Respondents were asked additional questions about RBC unit expiration, modification policies, and quality control practices. The median limit for RBC unit age was 25 days (IQR 14–42 days) for adults and 20 days (IQR 14–42 days) for children. Ten institutions (16%) reported a universal irradiation policy. A median expiration of 11 days (IQR 4–28 days) was used for irradiated units. No survey respondents washed RBC units, irradiated or non‐irradiated, beyond a certain number of days.

Thirteen percent of programs (8/62) test the hematocrit for each RBC unit used for RCE. Respondents commented that hematocrit was checked using the hematology analyzer. Only one respondent specified that a sample from each RBC unit was taken directly from the blood bag. Twenty‐four percent of programs (15/62) verified the mean RBC unit hematocrit with the blood supplier on a regular basis, though that timeframe varied.

## 4 Discussion

In this survey of apheresis practitioners, we summarized pre‐ and intraprocedural practices for patients with SCD receiving chronic RCE. Many details evaluated in this survey, such as IHD‐RCE parameters, are not specifically addressed in current society guidelines. Therefore, it is not surprising that we noted considerable variation in practices; though common themes were identified.

More than half of survey respondents reported using at least one fixed RCE parameter (i.e., FCR, end hematocrit, and/or HbS%), though no parameter was fixed for more than 50% of respondents. The end hematocrit was the most frequently fixed parameter, with a median value of 30%. This finding is consistent with the survey performed by Karafin et al. [10] In patients with SCD, peripheral oxygen delivery is thought to be optimized at a hematocrit of 30% [14, 15]. Accordingly, many pivotal SCD clinical trials used hematocrit values of 27%–36% as transfusion targets, and many societies have adopted these values for guideline recommendations [1, 3, 4, 16, 17]. Although a sustained hematocrit of 30% may represent a presumed “gold standard,” patients with low baseline hematocrits (e.g., less than 24%–27%) are at risk of developing systemic hemosiderosis with repeated targeting of this value. The ubiquitousness of this practice underscores the need for robust iron status monitoring and as‐needed chelation for all patients receiving chronic RCE [18].

Although not explicitly stated in current society guidelines, individualized FCR targets appear preferred to fixed FCR targets in apheresis centers that perform chronic RCE. A fixed FCR of 30% has historically been the default chronic RCE target [3, 4]. However, an FCR of 30% may be inappropriate to use for a patient with a low pre‐procedure HbS%, resulting in unnecessary RBC usage and donor exposures [19, 20, 21]. Personalization of FCR targets requires either availability of the pre‐procedure HbS% at the time of the procedure or prediction of the pre‐procedure HbS% based on prior values and has been shown to reduce RBC utilization [19]. Use of individualized FCR targets and increasing availability of pre‐procedure HbS% values could be recommended by societies such as ASFA and the Association for the Advancement of Blood and Biotherapies (AABB), as a low‐cost, high‐impact blood conservation strategy.

Information campaigns to increase awareness of appropriate priming with RCE may also spur donor RBC savings. For the Spectra Optia Apheresis System (Terumo BCT, Lakewood, CO), a custom fluid prime (e.g., plasma, 5% albumin, or RBCs) is recommended to prevent symptomatic hypovolemia or anemia when either greater than 10%–15% of the patient's TBV or greater than 10%–15% of the patient's total red cell volume (RCV) will be held in the extracorporeal circuit (185 mL) of the apheresis device [22]. The Spectra Optia Apheresis System handles custom priming with albumin and RBCs differently. In both cases, at the start of the procedure, the device draws the custom prime fluid into the inlet line, channel, reservoir, and return line. Following albumin prime, replacement RBCs mix with albumin in the return line until the replacement fluid mirrors the patient's pre‐procedure hematocrit. The replacement fluid flows into the patient immediately after starting the procedure to maintain their hematocrit, and the albumin present in the channel is diverted to the waste bag. By contrast, following RBC prime, the priming RBCs, including those in the channel, are transfused to the patient, transiently increasing the patient's hematocrit [23]. Neither priming method results in a decrease in patient hematocrit from the pre‐procedure value. Fifty‐three percent of adult‐only institutions reported using pre‐procedure hematocrit as a standalone indication to perform an RBC prime, even though significant worsening of anemia is unlikely in patients weighing more than 30 kg (most adults), and only 40% of respondents ever used an albumin prime, though this method reduces RBC utilization in patients eligible for priming and maintains the pre‐procedure hematocrit. These survey results suggest that there may be an opportunity to educate providers regarding non‐RBC prime options.

Many survey respondents perform IHD‐RCE to reduce donor exposures and iron loading. Kim et al. first described the benefits of replacing RBC volume with saline or autologous plasma at the beginning of the RCE procedure to maximize efficiency [7]. This hemodilution step was adapted for the COBE Spectra Apheresis System (Terumo BCT, Lakewood, CO), though several manual steps were required to perform the procedure [8]. Therefore, the operator had significant flexibility to select depletion and minimum hematocrit values. Following discontinuation of the COBE Spectra, the Spectra Optia Apheresis System was developed with a programmed IHD‐RCE protocol that constrains the minimum hematocrit range from 2% less than the pre‐procedure hematocrit to as low as 20%. Operators using IHD‐RCE can anticipate requiring one fewer RBC unit in adult patients to reach a given FCR or reaching a decreased FCR with the same number of RBC units for a standard RCE [24, 25, 26]. This procedure is an important, underutilized tool for blood conservation.

Though the majority of respondents in this survey reported using IHD‐RCE to reduce iron overload, no studies definitively support the assumption that IHD‐RCE reduces iron loading more than standard RCE [9, 26]. As mentioned above, the rate of iron loading may depend on thoughtful programming of the end hematocrit relative to the pre‐procedure hematocrit and patient adherence to iron chelation. It should be noted, however, that the impact of IHD‐RCE on iron status is poorly reported. Because reducing iron burden is a priority, future studies should seek to clarify the relationship between IHD‐RCE and systemic iron loading and describe the ideal parameters to induce iron loss.

Survey respondents utilize multiple strategies to cope with adverse symptoms associated with IHD‐RCE. Although limited data suggest similar adverse event rates with RCE with and without IHD‐RCE, prior investigators identified subgroups of patients who experienced more adverse effects than others, suggesting that some patients may be at unique risk for poorly tolerating IHD‐RCE [8, 24, 27]. Wade et al. specifically found a higher rate of IHD‐RCE‐associated adverse events in patients with a pre‐procedure hematocrit ≥ 30%, non‐hemoglobin SS or Sβ^0^ thalassemia genotypes, and a history of neurosurgical revascularization [27]. When symptoms occur, using 5% albumin instead of saline as the replacement fluid, increasing the minimum hematocrit, and providing additional pre‐procedure hydration have been suggested as strategies to prevent symptoms during subsequent procedures. Most survey respondents attempted multiple strategies prior to switching to standard RCE; however, a significant minority (29%) abandoned IHD‐RCE without trying any procedural modifications. Without further study, it is difficult to know if these institutions are unaware of strategies to reduce IHD‐RCE‐associated symptoms or have limited resources to reduce potential adverse events. Programs with a low threshold to discontinue IHD‐RCE or who have yet to adopt IHD‐RCE as a standard practice may benefit from additional published guidance on real‐world strategies to mitigate IHD‐RCE‐associated adverse effects.

It remains unclear which individual patient characteristics should preclude the use of IHD‐RCE. The 2020 ASH Sickle Cell Transfusion Guidelines suggest consultation with a hematologist and transfusion medicine specialist for individual risk determination when IHD‐RCE is considered and further advise avoidance of IHD‐RCE in patients with recent stroke or transient ischemic attack, severe vasculopathy, and severe cardiopulmonary disease, though these terms are not clearly defined [6]. Left ventricular ejection fraction less than 40% was cited as the most common reason for avoiding IHD‐RCE. This may be due to a lack of published experience using IHD‐RCE in patients with heart failure compared to those with cerebrovascular complications [24, 27]. Cardiovascular complications, such as pulmonary hypertension, hemosiderosis, and cardiomyopathy, have become leading causes of morbidity and mortality in patients with SCD and may be underdiagnosed in adult patients, including those receiving CTT [28, 29, 30]. Studies suggest that IHD‐RCE may cause modest reductions in blood pressure and increases in heart rate during the hemodilution phase [24, 26]. Although these vital sign changes produce no clinical change in most, they may be significant in individuals with tenuous cardiopulmonary status. Intentional evaluation for these comorbidities prior to initiation of or interval screening while receiving IHD‐RCE may be reasonable for risk stratification and to improve our understanding of how IHD‐RCE affects individuals with SCD‐associated cardiovascular disease.

Our survey showed that patients receiving chronic RCE often receive hydroxyurea for dual disease modification. These findings are consistent with the results reported by Karafin et al. [10] Recently, results of a pilot study evaluating the feasibility of combined hydroxyurea and chronic simple transfusions (NCT03644953) in pediatric patients suggest that the addition of hydroxyurea reduces RBC transfusion volume by increasing pre‐transfusion hemoglobin. Predictably, per‐protocol subjects demonstrated increased pre‐transfusion HbS%, though this may have been offset by concurrent production of hemoglobin F%, as subjects exhibited decreased hemolysis markers when compared to pre‐intervention values [31]. These findings require confirmation with large studies, though they provide support for this already widespread practice. Coadministration of hydroxyurea may also benefit patients by reducing circulating neutrophils and expression of cell adhesion proteins, factors not addressed by RCE or simple transfusion therapy but that may alter the heightened inflammatory state associated with SCD [32, 33, 34]. Additional reporting of institutional experiences with combined hydroxyurea and CTT may improve our understanding of optimal timing and dosage of this medication to improve patient outcomes and RBC savings.

This survey had a few limitations. First, inconsistencies in survey responses suggested that some survey questions may have benefited from clarification. For example, a small group of respondents reported using fixed FCR and HbS% goals; however, these values are codependent. Further information would help determine whether respondents targeted fixed pre‐ or post‐procedure HbS% goals and if or how these values informed FCR targets. Secondly, the survey was predominantly completed by ASFA members from the United States, although an attempt was made to encourage survey participation by international ASFA members and non‐ASFA affiliated professionals who perform apheresis. We acknowledge that many apheresis services are provided by third parties, nephrologists, and hematologists who may not have been captured in this survey, but whose practices would be of interest to describe. International institutions may approach the application of RCE and blood conservation in novel ways that would be valuable to capture. Lastly, because the survey sought to evaluate RCE practices in SCD, survey respondents may disproportionately represent centers with sizeable SCD populations. Thus, the practice patterns described here, such as prevalence of IHD‐RCE use, may not be representative of all apheresis providers.

## 5 Conclusions

In summary, we performed a survey of RCE practices among apheresis providers and found increased implementation of strategies to reduce donor exposure and conserve RBC units (e.g., individualized FCR, IHD‐RCE) compared to the results of the 2018 ASFA RCE practices survey. However, we also found many areas where further provider education or research is needed to optimize the safety and efficacy of RCE practice, including IHD‐RCE. Consensus on best practice in RCE among clinical and apheresis providers may be the first step towards achieving these goals.

## Funding

The authors have nothing to report.

## Ethics Statement

Ethical approval was not required for this study as the research was determined to be exempt by the Children's National Hospital Institutional Review Board on 8/24/2023.

## Consent

Informed consent was not required for this study as the research was determined by the Children's National Hospital Institutional Review Board not to involve human subjects as defined by DHHS and FDA regulations.

## Conflicts of Interest

The authors declare no conflicts of interest.

## Supporting information


**Table S1:** Chronic Red Cell Exchange Survey.
**Table S2:** Characteristics of respondents (*n* = 62).
**Table S3:** Likelihood to set the RCE parameters based on respondent characteristics (OR, 95% CI, Univariate).
**Table S4:** Use of sickle‐cell specific disease modifying therapies concurrent with RCE.

## Data Availability

The data that support the findings of this study are available from the corresponding author upon reasonable request.

## References

[1] R. J. Adams , V. C. McKie , L. Hsu , et al., “Prevention of a First Stroke by Transfusions in Children With Sickle Cell Anemia and Abnormal Results on Transcranial Doppler Ultrasonography,” New England Journal of Medicine 339, no. 1 (1998): 5–11, 10.1056/NEJM199807023390102. 9647873

[2] R. E. Ware , W. H. Schultz , N. Yovetich , et al., “Stroke With Transfusions Changing to Hydroxyurea (SWiTCH): A Phase III Randomized Clinical Trial for Treatment of Children With Sickle Cell Anemia, Stroke, and Iron Overload,” Pediatric Blood & Cancer 57, no. 6 (2011): 1011–1017, 10.1002/pbc.23145. 21826782 PMC3171640

[3] L. Connelly‐Smith , C. R. Alquist , N. A. Aqui , et al., “Guidelines on the Use of Therapeutic Apheresis in Clinical Practice – Evidence‐Based Approach From the Writing Committee of the American Society for Apheresis: The Ninth Special Issue,” Journal of Clinical Apheresis 38, no. 2 (2023): 77–278, 10.1002/jca.22043. 37017433

[4] “Evidence‐Based Management of Sickle Cell Disease: Expert Panel Report, 2014 | NHLBI, NIH,” accessed March 11, 2025, https://www.nhlbi.nih.gov/health‐topics/evidence‐based‐management‐sickle‐cell‐disease.

[5] S. T. Chou and R. M. Fasano , “Management of Patients With Sickle Cell Disease Using Transfusion Therapy,” Hematology/Oncology Clinics of North America 30, no. 3 (2016): 591–608, 10.1016/j.hoc.2016.01.011. 27112998

[6] S. T. Chou , M. Alsawas , R. M. Fasano , et al., “American Society of Hematology 2020 Guidelines for Sickle Cell Disease: Transfusion Support,” Blood Advances 4, no. 2 (2020): 327–355, 10.1182/bloodadvances.2019001143. 31985807 PMC6988392

[7] H. Kim , N. Dugan , J. Silber , et al., “Erythrocytapheresis Therapy to Reduce Iron Overload in Chronically Transfused Patients With Sickle Cell Disease,” Blood 83, no. 4 (1994): 1136–1142, 10.1182/blood.V83.4.1136.1136. 8111053

[8] R. Sarode , K. Matevosyan , Z. R. Rogers , J. D. Burner , and C. Rutherford , “Advantages of Isovolemic Hemodilution‐Red Cell Exchange Therapy to Prevent Recurrent Stroke in Sickle Cell Anemia Patients,” Journal of Clinical Apheresis 26, no. 4 (2011): 200–207, 10.1002/jca.20294. 21786315

[9] B. Nourse , M. Reddy , J. Jones , and M. H. Tran , “Benefits of Isovolemic Hemodilution Red Cell Exchange: Real or Imagined?,” Transfusion (Paris) 66, no. 4 (2026): 763–769, 10.1111/trf.70166. 41821357

[10] M. S. Karafin , J. E. Hendrickson , H. C. Kim , et al., “Red Cell Exchange for Patients With Sickle Cell Disease: An International Survey of Current Practices,” Transfusion (Paris) 60, no. 7 (2020): 1424–1433, 10.1111/trf.15863. 32583456

[11] P. A. Harris , R. Taylor , R. Thielke , J. Payne , N. Gonzalez , and J. G. Conde , “Research Electronic Data Capture (REDCap)—A Metadata‐Driven Methodology and Workflow Process for Providing Translational Research Informatics Support,” Journal of Biomedical Informatics 42, no. 2 (2009): 377–381, 10.1016/j.jbi.2008.08.010. 18929686 PMC2700030

[12] P. A. Harris , R. Taylor , B. L. Minor , et al., “The REDCap Consortium: Building an International Community of Software Platform Partners,” Journal of Biomedical Informatics 95 (2019): 103208, 10.1016/j.jbi.2019.103208. 31078660 PMC7254481

[13] “Pfizer Voluntarily Withdraws All Lots of Sickle Cell Disease Treatment OXBRYTA (voxelotor) From Worldwide Markets | Pfizer,” accessed August 2, 2025, https://www.pfizer.com/news/press‐release/press‐release‐detail/pfizer‐voluntarily‐withdraws‐all‐lots‐sickle‐cell‐disease.

[14] K. Jan , S. Usami , and J. A. Smith , “Effects of Transfusion on Rheological Properties of Blood in Sickle Cell Anemia,” Transfusion (Paris) 22, no. 1 (1982): 17–20, 10.1046/j.1537-2995.1982.22182154208.x. 7064201

[15] S. Chien , S. Usami , and J. F. Bertles , “Abnormal Rheology of Oxygenated Blood in Sickle Cell Anemia,” Journal of Clinical Investigation 49, no. 4 (1970): 623–634, 10.1172/JCI106273. 5443167 PMC322516

[16] M. R. DeBaun , M. Gordon , R. C. McKinstry , et al., “Controlled Trial of Transfusions for Silent Cerebral Infarcts in Sickle Cell Anemia,” New England Journal of Medicine 371, no. 8 (2014): 699–710, 10.1056/NEJMoa1401731. 25140956 PMC4195437

[17] M. R. DeBaun , L. C. Jordan , A. A. King , et al., “American Society of Hematology 2020 Guidelines for Sickle Cell Disease: Prevention, Diagnosis, and Treatment of Cerebrovascular Disease in Children and Adults,” Blood Advances 4, no. 8 (2020): 1554–1588, 10.1182/bloodadvances.2019001142. 32298430 PMC7189278

[18] J. M. Ross , S. Forté , N. Mercure‐Corriveau , et al., “Automated Red Blood Cell Exchange With a Post‐Procedure Haematocrit Targeted at 34% in the Chronic Management of Sickle Cell Disease,” British Journal of Haematology 205, no. 4 (2024): 1556–1564, 10.1111/bjh.19674. 39081092

[19] S. Uter , H. H. An , G. E. Linder , et al., “Measures to Reduce Red Cell Use in Patients With Sickle Cell Disease Requiring Red Cell Exchange During a Blood Shortage,” Blood Advances 5, no. 12 (2021): 2586–2592, 10.1182/bloodadvances.2021004395. 34152394 PMC8270657

[20] K. R. Buban , C. E. Lawrence , X. J. Zhu , et al., “Algorithm‐Based Selection of Automated Red Blood Cell Exchange Procedure Goals Reduces Blood Utilization in Chronically Transfused Adults With Sickle Cell Disease,” Journal of Clinical Apheresis 37, no. 5 (2022): 468–475, 10.1002/jca.22004. 36053868

[21] J. M. Jones , A. D. Swett , E. P. Crowe , C. Lawrence , E. M. Bloch , and S. M. Lanzkron , “Implementation of National Blood Conservation Recommendations at an Adult Sickle Cell Center,” Transfusion (Paris) 62, no. 9 (2022): 1763–1771, 10.1111/trf.17007. 35837727

[22] “Spectra Optia Apheresis System Operator's Manual”.

[23] Terumo BCT, Inc ., “Red Blood Cell Exchange (V12 RBCX) Rx Only Procedure Training,” (2023) Document Number: TS‐OPTI‐00799.

[24] Y. Ziemba , C. Xu , K. M. Fomani , et al., “Safety and Benefits of Automated Red Cell Depletion‐Exchange Compared to Standard Exchange in Patients With Sickle Cell Disease Undergoing Chronic Transfusion,” Transfusion (Paris) 61, no. 2 (2021): 526–536, 10.1111/trf.16225. 33368343

[25] K. Uminski , I. Perelman , A. T. Tinmouth , and J. Mack , “Use of Automated Isovolemic Hemodilution Red‐Cell Exchange in Patients With Sickle Cell Disease: A Canadian Single Center Experience,” Transfusion (Paris) 65, no. 2 (2025): 325–332, 10.1111/trf.18119. 39950204

[26] M. S. Reddy , A. Alkashash , A. Nord , and A. Tetrick , “Revisiting the Benefits of Isovolemic Hemodilution Red Cell Exchange for Sickle Cell Disease,” Journal of Clinical Apheresis 38, no. 5 (2023): 522–528, 10.1002/jca.22054. 37092306

[27] J. Wade , M. E. M. Yee , K. A. Easley , et al., “Procedural Adverse Events in Pediatric Patients With Sickle Cell Disease Undergoing Chronic Automated Red Cell Exchange,” Transfusion (Paris) 62, no. 3 (2022): 584–593, 10.1111/trf.16807. PMC8959247 35072269

[28] C. D. Fitzhugh , N. Lauder , J. C. Jonassaint , et al., “Cardiopulmonary Complications Leading to Premature Deaths in Adult Patients With Sickle Cell Disease,” American Journal of Hematology 85, no. 1 (2010): 36–40, 10.1002/ajh.21569. 20029950 PMC3865703

[29] N. Hammoudi , F. Lionnet , A. Redheuil , and G. Montalescot , “Cardiovascular Manifestations of Sickle Cell Disease,” European Heart Journal 41, no. 13 (2020): 1365–1373, 10.1093/eurheartj/ehz217. 31005979

[30] T. Simon , L. Savale , K. Grundtvig Skaarup , et al., “Sickle Cell Diastolic Cardiomyopathy and Mortality Risk: A Novel Echocardiographic Framework for Prognostic Stratification,” American Journal of Hematology 100, no. 11 (2025): 1940–1951, 10.1002/ajh.27768. 40693500 PMC12516662

[31] R. S. Nickel , S. Margulies , K. Panchapakesan , et al., “Adding Hydroxyurea to Chronic Transfusion Therapy for Sickle Cell Anemia Reduces Transfusion Burden,” Transfusion (Paris) 65, no. 1 (2025): 38–49, 10.1111/trf.18073. PMC11750600 39580793

[32] G. M. Lee , K. Boyle , M. Batchvarova , et al., “Red cell exchange modulates neutrophil degranulation responses in sickle cell disease,” Transfusion (Paris) 64, no. 9 (2024): 1752–1761, 10.1111/trf.17947. 38979976

[33] N. Yurtsever , N. Tong , S. Geetha , V. Nandi , and P. A. Shi , “Post‐Exchange Neutrophil Count, but Not Post‐Hematocrit, Predicts Endogenous Erythropoiesis in Patients With Sickle Cell Disease Undergoing Chronic Red Cell Exchange,” Transfusion (Paris) 64, no. 12 (2024): 2270–2278, 10.1111/trf.18044. 39404130

[34] A. K. Dembélé , P. Hermand , F. Missud , et al., “Persistence of Chronic Inflammation After Regular Blood Transfusion Therapy in Sickle Cell Anemia,” Blood Advances 7, no. 3 (2023): 309–313, 10.1182/bloodadvances.2022007464. 35834752 PMC9898595

