# Supplementary material for: Variation in Chronic Automated Red Cell Exchange Practices for Sickle Cell Disease: Insights Into Isovolemic Hemodilution Use
Source: J Clin Apher. 2026 Jun 18;41(3):e70146. doi: 10.1002/jca.70146 (PMC13280177; doi:10.1002/jca.70146)

Supplemental Data

Supplemental Table 1

Chronic Red Cell Exchange Survey

Instructions: The intent of this survey is to better understand how providers practice chronic red cell exchange in patients with sickle cell disease, specifically in patients with HbSS or HbS/B-zero thalassemia. Ideally this survey should be completed by an apheresis MD/DO in concert with the hematology team. We would prefer if each institution submits a single response. A .pdf of all of the questions was attached to the email inviting you to participate in this survey. If necessary, questions can be skipped and the survey submitted without answering all questions. This survey is expected to take 10-20 minutes to complete. You can save the survey and come back to it later.

Please complete the survey below. Thank you!

|  | Approximately, how many total patients with sickle cell anemia (SS, SB0) do you currently have on chronic red cell exchange? | [free text] |
| --- | --- | --- |
|  | What age of patients do you treat? | Adult (>18yo)  Pediatric (< or = 18yo)  Both |
| Setting Parameters for Chronic Red Cell Exchange | | |
|  | Do you have set chronic red cell exchange goals (example: FCR or hematocrit) used for all patients? | Yes  No |
|  | Which chronic red cell exchange parameters are set/fixed for all patients? Check all that apply | FCR  Target HCT  Target HbS%  Other |
| 5. | What is your set/fixed FCR? | [free text] |
| 6. | What is your set/fixed target HCT (in %)? | [free text] |
| 7. | What is your set/fixed target HbS%? | [free text] |
| 8. | If other, please describe | [free text] |
| 9. | What labs do you typically obtain prior to each red cell exchange? Check all that apply | CBC  Hematocrit (if no CBC obtained)  Reticulocyte count  Ferritin  HbS%  Other |
| 10. | If other, please described | [free text] |
| 11. | Do you take the results of the pre-red cell exchange Hb fraction/electrophoresis into account to set the FCR goal for that exchange? | Yes  No |
| 12. | Do you use the pre-red cell exchange Hb fraction/electrophoresis to adjust the FCR for subsequent procedures? | Yes  No |
| 13. | What is the turn-around-time for your Hb fraction/electrophoresis results? | Less than or equal to 3 days  4-7 days  Greater than 7 days |
| 14. | Do you take the results of the pre-red cell exchange Hb fraction/electrophoresis into consideration to adjust the interval between procedures? | Yes  No |
| 15. | Within how many days before the procedure do you collect the sample for Hb fraction/electrophoresis? | [free text] |
| 16. | Do you take the results of the pre-red cell exchange HCT into account when setting the end HCT target of the red cell exchange? | Yes  No |
| 17. | Within how many days before the procedure do you collect the sample for HCT? | [free text] |
| 18. | Do you take the results of the reticulocyte count into account to set the end HCT of the red cell exchange? | Yes  No |
| 19. | Within how many days before the procedure do you collect the sample for reticulocyte count? | [free text] |
| 20. | What criteria do you use to decide whether to custom prime with red cells? Check all that apply. | Weight  Age  %Extracorporeal Volume  Pre-procedural HCT  Clinical Stability  We do not custom prime with red cells for red cell exchange procedures |
| 21. | What criteria do you use to decide whether to custom prime with albumin? Check all that apply. | Weight  Age  %Extracorporeal Volume  Pre-procedural HCT  Clinical Stability  We do not custom prime with albumin for red cell exchange procedures |
| 22. | Do you test the HCT of each unit used for red cell exchange? | Yes  No |
| 23. | Do you verify, on a regular basis, that the product HCT entered is accurate (e.g. confirm with the blood supplier)? | Yes  No |
| 24. | If yes, what is your protocol for verifying (frequency, method, etc.)? | [free text] |
| 25. | What labs for you typically obtain immediately after red cell exchange? Check all that apply. | Hb fraction/electrophoresis to verify target HbS% was achieved  HCT to verify taget end HCT was achieved  Other  We do not obtain post exchange labs routinely |
| 26. | If other, please describe | [free text] |
| 27. | In a patient with no history of alloantibodies and no history of positive antibody screen, do you perform an electronic crossmatch? | Yes  No |
| Depletion-Exchange or Isovolemic Hemodilution (IVH) Exchange*  Compared to standard red cell exchange, this modification can either lower the number of units needed to achieve a given FCR or lower the FCR for a given number of units. The goal of these questions is to study safety concerns around depletion-exchange. | | |
| 28. | Do you perform depletion-exchange (isovolemic hemodilution exchange)? | Yes  No |
| 29. | What are the reasons(s) you perform depletion exchange (IVH)? Check all that apply | To save money using fewer red cell units  To reduce donor exposures by using fewer red cell units  To lower FCR for a given number of units and increase time between procedures  To decrease iron overload  To decrease procedure time  Other |
| 30. | If other, please describe. | [free text] |
| 31. | For depletion-exchange (IVH), how do you determine the minimum HCT for the depletion phase? Check all that apply. | Absolute lower limit (e.g. regardless of starting HCT, would decrease to 21% at nadir)  Relative % drop from baseline HCT (e.g. drop HCT by 25%, so starting HCT 30%, would decrease to 22.5% at nadir)  Absolute decrease from baseline HCT (e.g. drop by 3%, so starting HCT 30%, would decrease to 27% at nadir)  Other |
| 32. | If other, please describe | [free text] |
| 33. | Specify your lower limit HCT, if applicable | [free text] |
| 34. | Specify your maximum relative % drop from baseline HCT, if applicable | [free text] |
| 35. | Specify your maximum absolute % decrease from baseline HCT, if applicable | [free text] |
| 36. | Of the following adult patients, for which, if any, would you NOT do depletion-exchange (IVH)? Check all that apply | We don’t treat adults with IVH  An adult with Moya Moya syndrome  An adult with silent infarcts on brain MRI  An adult with mean pulmonary artery pressure > 25 mmHg  An adult with left ventricular ejection fraction < 40%  An adult with GFR dysfunction < 60 mL/min/1.73 m2  Other  Would consider all of these patients eligible for depletion-exchange |
| 37. | If other, please describe | [free text] |
| 38. | What is the earliest time point (in days) after an overt stroke that chronic use of depletion-exchange (IVH) would be considered in an adult patient? | [free text] |
| 39. | Of the following pediatric patients, for which, if any, would you NOT perform depletion-exchange (IVH)? Select all that apply. | We don’t treat children with IVH  A child with Moya Moya syndrome  A child with silent infarcts on brain MRI  A child with mean pulmonary artery pressure > 25 mmHg  A child with left ventricular ejection fraction < 40%  A child with GFR dysfunction < 60 mL/min/1.73 m2  Other  Would consider all of these patients eligible for depletion exchange |
| 40. | If other, please describe | [free text] |
| 41. | What is the earliest time point (in days) after an overt stroke that chronic use of depletion-exchange (IVH) would be considered in a pediatric patient? | [free text] |
| 42. | If a patient experiences clinically relevant hypotension or clinical symptoms of anemia during depletion0exchange (IVH), and citrate toxicity was ruled out, what would your corrective action be (for further procedures) if you felt that changes were indicated? Check all that apply | Modify depletion parameters by increasing nadir hematocrit  Try 5% albumin or 6% hydroxyethylstarch (HES) instead of saline replacement  Switch to standard exchange (without depletion)  Provide additional hydration prior to subsequent procedures  No changes  Other |
| 43. | If other, please describe | [free text] |
| If you would attempt multiple interventions, please rank which order you would perform them in. | | |
| 44. | Modify depletion parameters would be your: | First option  Second option  Third option  Fourth option  Fifth option  Sixth option |
| 45. | Trying 5% albumin or 6% hydroxyethylstarch (HES) instead of saline replacement would be your: | First option  Second option  Third option  Fourth option  Fifth option  Sixth option |
| 46. | Switch to standard exchange (without depletion) would be your: | First option  Second option  Third option  Fourth option  Fifth option  Sixth option |
| 47. | Provide additional hydration prior to subsequent procedures would be your: | First option  Second option  Third option  Fourth option  Fifth option  Sixth option |
| 48. | No changes would be your: | First option  Second option  Third option  Fourth option  Fifth option  Sixth option |
| 49. | Your other option would be your: | First option  Second option  Third option  Fourth option  Fifth option  Sixth option |
| Red Cell Unit Modifiers of Hematocrit Maintenance Between Red Cell Exchange Procedures  The goal of these questions is to determine whether data is available to study whether red cell unit characteristics influence the maintenance hematocrit between procedures. | | |
| 50. | In adults, what is your institution’s age limit (in days) for red cells used in chronic red cell exchange procedures? If your institution has no limit, select 42 days | [free text] |
| 51. | In children, what is your institution’s age limit (in days) for red cells used in chronic red cell exchange procedures? If your institution has no limit, select 42 days | [free text] |
| 52. | Does your institution have a universal (for all patients) irradiation policy? | Yes  No |
| 53. | For patients with sickle cell disease, how many days post-irradiation will you use a unit? | [free text] |
| 54. | For patients with sickle cell disease, do you wash red cells that are past a certain RBC storage age? | Yes  No |
| 55. | After what storage age (in days) do you wash an RBC unit? | [free text] |
| 56. | For patients with sickle cell disease, do you wash red blood cells that are beyond a certain number of days post irradiation? | Yes  No |
| 57. | How many days after irradiation do you wash a RBC unit? | [free text] |
| 58. | Do you test the HCT of washed units used for red cell exchange? | Yes  No |
| Other Medications Used in Patients Receiving Red Cell Exchange  The goal of these questions is to determine whether data is available to study red cell exchange used in conjunction with other medications used to treat sickle cell disease. | | |
| 59. | Approximately what percent (%) of your sickle cell patient population is on concomitant hydroxyurea? | [free text] |
| 60. | What are your reason(s) for using hydroxyurea? Check all that apply. | N/A, do not use hydroxyurea  Decrease WBC or neutrophil count  Increase HbF%  Decrease hemolysis  Increase hemoglobin  Decrease RBC replacement volumes needed for exchange  To eventually stop red cell exchange/transfusions  Continued from prior  Decrease vasoocclusive events  Decrease platelet count  Other |
| 61. | If other, please describe | [free text] |
| 62. | Approximately what percent (%) of your sickle cell patient population is on concomitant voxelotor? | [free text] |
| 63. | What are your reasons(s) for using voxelotor? Check all that apply. | N/A, do not use voxelotor  Decrease hemolysis  Increase hemoglobin  Decrease RBC replacement volumes needed for exchange  To eventually stop red cell exchange/transfusions  Continued from prior  Decrease vasoocclusive events  Other |
| 64. | If other, please describe | [free text] |
| 65. | Approximately what percent (%) of your sickle cell patient population is on concomitant crizanlizumab? | [free text] |
| 66. | What are your reasons(s) for using crizanlizumab? Select all that apply. | N/A, do not use crizanlizumab  Decrease hemolysis  Increase hemoglobin  Decrease vasoocclusive events  Continued from prior  Improved quality of life  Other |
| 67. | If other, please describe | [free text] |
| Demographic Information of Individual Completing the Survey | | |
| 68. | Optional: What institution are you from? | [free text] |
| 69. | As part of your clinical responsibilities, are you directly responsible for setting red cell exchange parameter targets? | Yes  No |
| 70. | How many years have you directly cared for patients with sickle cell disease on chronic red cell exchange? | 0-3  4-6  7-10  11-15  16-20  >20 |
| 71. | Are you the director of the apheresis service at your institution? | Yes  No |
| 72. | Are you the director of the sickle cell clinic at your institution? | Yes  No |

*Isovolemic hemodilution is abbreviated “IVH” in the distributed survey. Since this is a non-standard acronym, isovolemic hemodilution is abbreviated “IHD-RCE,” a more commonly used abbreviation, in the manuscript main text for easier readability.


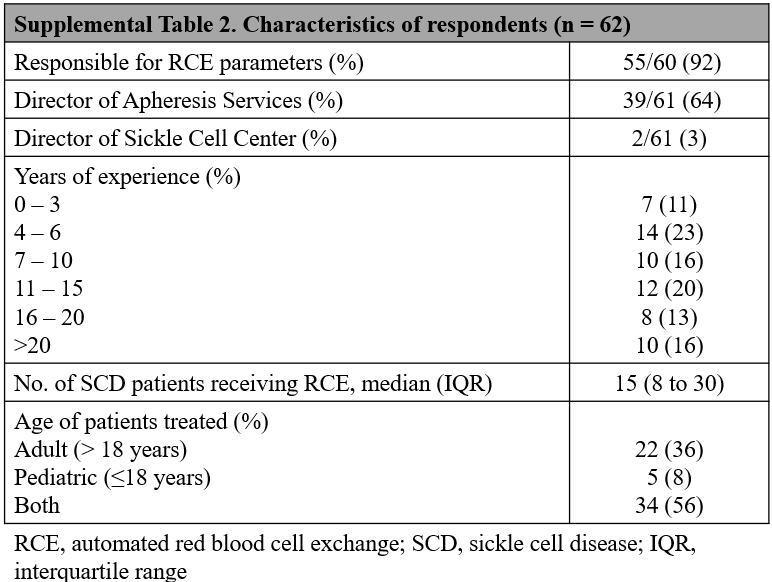


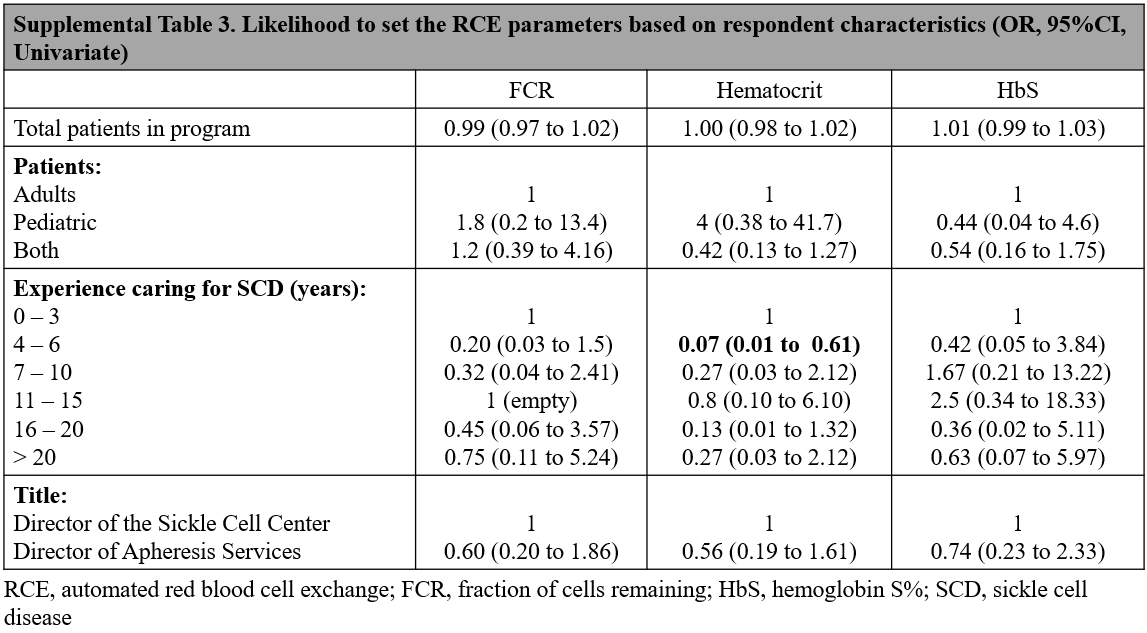


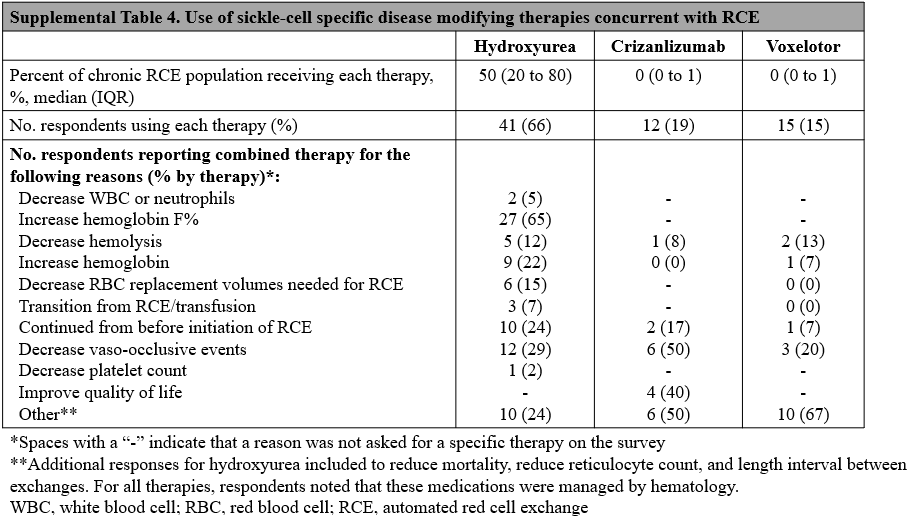

Supplement: Supplementary file 1 — Table S1: Chronic Red Cell Exchange Survey. Table S2: Characteristics of respondents (n = 62). Table S3: Likelihood to set the RCE parameters based on respondent characteristics (OR, 95% CI, Univariate). Table S4: Use of sickle‐cell specific disease modifying therapies concurrent with RCE. [file JCA-41-e70146-s001.docx]
